# Genome-Wide Identification and Functional Analysis of Nitrate Transporter Genes (*NPF*, *NRT2* and *NRT3*) in Maize

**DOI:** 10.3390/ijms241612941

**Published:** 2023-08-18

**Authors:** Lihua Jia, Desheng Hu, Junbo Wang, Yuanyuan Liang, Fang Li, Yi Wang, Yanlai Han

**Affiliations:** 1 State Key Laboratory of Wheat and Maize Crop Science, College of Resources and Environment, Henan Agricultural University, Zhengzhou 450046, China; jlhhena@163.com (L.J.); desheng1025@126.com (D.H.); 13460753905@163.com (J.W.); 18338769708@163.com (Y.L.); fangli0901@henau.edu.cn (F.L.); 2 College of Agronomy, Henan Agricultural University, Zhengzhou 450046, China

**Keywords:** nitrate transporters, bioinformatic analysis, nitrogen efficiency, nitrate uptake rates

## Abstract

Nitrate is the primary form of nitrogen uptake in plants, mainly transported by nitrate transporters (NRTs), including NPF (NITRATE TRANSPORTER 1/PEPTIDE TRANSPORTER FAMILY), NRT2 and NRT3. In this study, we identified a total of 78 *NPF*, seven *NRT2*, and two *NRT3* genes in maize. Phylogenetic analysis divided the *NPF* family into eight subgroups (*NPF1*-*NPF8*), consistent with the results in *Arabidopsis thaliana* and rice. The *NRT2* family appears to have evolved more conservatively than the *NPF* family, as *NRT2* genes contain fewer introns. The promoters of all *NRTs* are rich in *cis*-acting elements responding to biotic and abiotic stresses. The expression of *NRTs* varies in different tissues and developmental stages, with some *NRTs* only expressed in specific tissues or developmental stages. RNA-seq analysis using Xu178 revealed differential expression of *NRTs* in response to nitrogen starvation and nitrate resupply. Moreover, the expression patterns of six key *NRTs* genes (*NPF6.6*, *NPF6.8*, *NRT2.1*, *NRT2.5* and *NRT3.1A/B*) varied in response to alterations in nitrogen levels across distinct maize inbred lines with different nitrogen uptake rates. This work enhances our understanding of the structure and expression of *NRTs* genes, and their roles in nitrate response, paving the way for improving maize nitrogen efficiency through molecular breeding.

## 1. Introduction

Nitrogen is an essential element for plant growth, metabolism and heredity, and its nutritional status directly affects crop yield. While the application of nitrogen fertilizer increases crop yield, it also gives rise to a range of environmental issues. Exploring the genetic potential of nitrogen use efficiency (NUE) in crops represents one effective strategy for improving NUE and reducing nitrogen fertilizer application rates. Plant NUE is mainly affected by N uptake efficiency (NUpE), N utilization (assimilation) efficiency (NUtE), N transport efficiency, and N remobilization efficiency [1]. In general, NUpE and NUtE are the main contributors to plant NUE. Nitrate transporters (NRTs) play a crucial and extensive role in uptaking nitrate from the soil and transporting it to different organs for plant growth and development. An in-depth analysis of *NRTs* genes can advance our understanding of the mechanism behind efficient nitrogen utilization and help in identifying NUE genes.

*Arabidopsis thaliana* and rice (*Oryza sativa*) possess 53 and 93 *NPF* genes respectively [2,3], the majority of which are low-affinity nitrate transporters except for *AtNPF6.3 (NRT1.1/CHL1)*, which has double affinity for nitrate uptake [4]. NRT2 family, in contrast to the NPF family, has seven and five members in *Arabidopsis thaliana* and rice, respectively [2], with the majority exhibiting high-affinity activity. *NPF* gene family members have diverse and pivotal roles in nitrate uptake, transportation and distribution within plants [2]. For example, *AtNPF6.3/NRT1.1* and *OsNPF6.5/NRT1.1B* are responsible for nitrate uptake in *Arabidopsis thaliana* and rice [5,6,7,8], while seven genes (*AtNPF7.3/NRT1.5*, *AtNPF7.2/NRT1.8*, *AtNPF2.9/NRT1.9*, *AtNPF2.3*, *OsNPF2.2*, *OsNPF2.4* and *OsNPF6.5/NRT1.1B)* are involved in the nitrate transportation from root to shoot [9,10,11,12,13,14]. *AtNPF1.1/NRT1.11*, *AtNPF1.2/NRT1.12* and *AtNPF2.13/NRT1.7* genes participate in the remobilization of nitrate from source to sink [1]. Unlike the NPF family, the functional activity of NRT2 transporters often depends on assistance from the ancillary proteins NAR2 (or NRT3) interacting with NRT2 [15]. Seven *NRT2* genes, including *AtNRT2.1*, *AtNRT2.2*, *AtNRT2.4*, *AtNRT2.5*, *OsNRT2.1*, *OsNRT2.2*, and *OsNRT2.4*, are involved in nitrate uptake in *Arabidopsis thaliana* and rice. Furthermore, *AtNRT2.4* and *AtNRT2.5* are also involved in the remobilization of nitrate from source to sink. Genetic improvement of some of these *NPF* and *NRT2* genes, such as *OsNRT1.1B* [7], *OsNRT2.3b* [16], *OsNPF6.1^HapB^* [17] and *OsNRT2.3a* [18], can significantly increase crop yield and NUE, highlighting the crucial roles of these genes in nitrate uptake and transport in plants.

Although extensive research in *Arabidopsis thaliana* and rice has been conducted, studies on the *NRTs* gene family in maize have primarily focused on analyzing their expression patterns and conducting electrophysiological studies of selected *NRTs*. For instance, Garnett T. et al. (2013) and Dechorgnat J. et al. (2019) investigated tissue-specific and nitrogen-linked expression profiles of certain nitrate transporters [19,20]. Furthermore, ZmNPF6.4 and ZmNPF6.6 have been modified to exhibit different selectivity to nitrate and chloride, with ZmNPF6.6 acting as a pH-dependent non-biphasic high-affinity nitrate-specific transporter [21]. NAR2.1/NRT2.1 has improved functional interaction in nitrate uptake along the root axis of maize, as evidenced by gene expression results [22]. Although *NPF* genes had been identified and named as family members based on version 3 of the maize B73 genome, phylogenetic analysis, gene structure and *cis*-elements have yet to be studied in-depth [3]. Additionally, there have been no studies exploring the contribution of key *NPF* or *NRT2* genes to plant NUE. Hence, in our study, we identified and characterized all members of the *NPF*, *NRT2* and *NRT3* gene family based on the maize B73 version 5 genome. We conducted RNA-seq analysis to investigate the expression patterns of all *NRTs* genes in response to changes in external nitrate levels. Based on the RNA-seq results, we identified six *NRTs* genes that were responsive to nitrate and potentially linked to nitrate uptake in maize. We subsequently examined the expression patterns of these candidate genes in four maize inbred lines with differing nitrogen uptake efficiency using quantitative real-time RT-PCR (RT-qPCR). Through the above work, we aim to obtain a comprehensive understanding of *NRTs* in maize, which will provide valuable information and serve as a reference for identifying key *NRTs* that control nitrate uptake and transport in maize.

## 2. Results

### 2.1. Identification of NPF, NRT2 and NRT3 Genes in Maize

A previous study named 79 *NPF* genes in maize based on the phylogenetic relationship of *NPF* genes in 31 fully sequenced plant genomes [3]. However, this analysis was conducted based on the V3 version of the B73 genome, which has since been updated to version V5 in 2021 with improved sequence accuracy and gene annotations. In this study, we identified 78 *NPF* genes (Table S1) in the maize B73 V5 genome by aligning all protein sequences to those of the 53 *NPF* genes in *Arabidopsis thaliana* and filtering for the PTR2 domain (Section 4). Two new *NPF* genes were identified, and four previously identified *NPF* genes were eliminated. Furthermore, one *NPF* gene identified previously was annotated as two adjacent genes in the maize B73 V5 genome. The nomenclature of *NPF* genes from the previous study was used for the commonly identified genes, while the newly identified *NPF* genes were named based on their subfamily, inferred from a tree constructed using all the protein sequences of *NPF* genes in maize and *Arabidopsis thaliana*. In addition, we identified seven *NRT2* genes and two *NRT3* genes in the maize B73 V5 genome using the same approach. The names of five *NRT2* genes and two *NRT3* genes provided in MaizeGDB were adopted, whereas the remaining *NRT2* genes were named *NRT2.4* and *NRT2.6*, respectively.

### 2.2. Phylogenetic Analysis of NPF, NRT2 and NRT3 Genes

**Figure 1:**
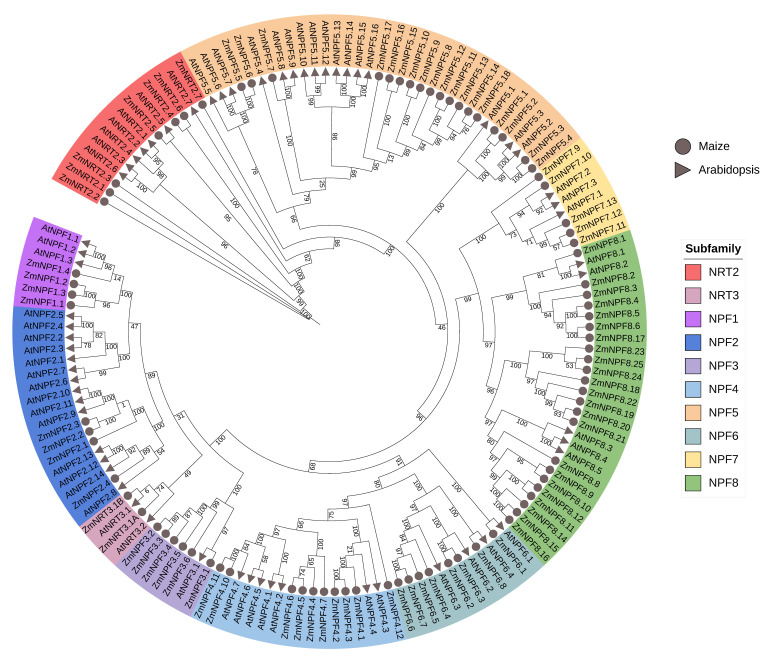
Phylogenetic tree of *NRTs* from maize and *Arabidopsis thaliana*. The unrooted neighbor-joining (NJ) tree was constructed using MEGA7.0 with 1000 bootstrap replicates. Rings of subtrees are colored, indicating different *NRTs* subgroups.

Being present in many plants including *Arabidopsis thaliana* [3], *NPF* genes can be categorized into eight subfamilies based on their protein sequences. To determine the phylogenetic relationships of *NPF*, *NRT2* and *NRT3* genes in maize, we constructed a phylogenetic tree (as outlined in Section 4). Our results revealed that members of the same subfamily in both maize and *Arabidopsis thaliana* were clustered together (Figure 1). *NRT2* genes formed a distinct clade from *NPF* and *NRT3* genes, while *NRT3* genes were clustered together with the *NPF1* and *NPF2* subfamilies.

### 2.3. Chromosomal Localization of NPF, NRT2 and NRT3 Genes

**Figure 2:**
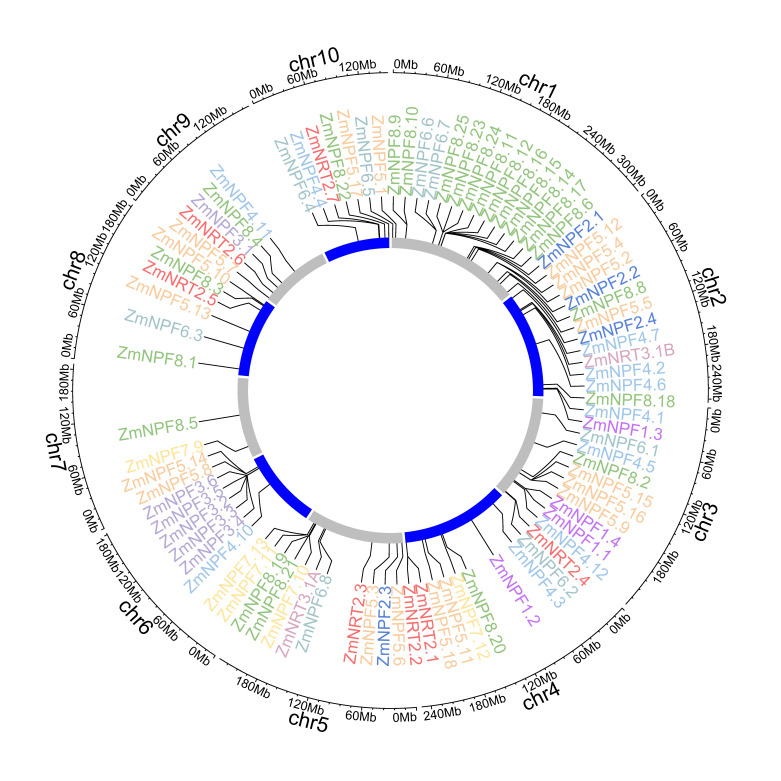
Chromosomal location of *NRTs* genes. Members of different subfamilies are indicated with varying colors.

A total of 87 *NRTs* genes were observed to be unevenly distributed across all ten chromosomes, with most genes tending to be localized toward the distal regions (Figure 2). Among the ten chromosomes, chromosome 1 possessed the greatest number of *NRTs* genes, with 23 genes, followed by chromosomes 3 and 5, each with 11 genes. On the other hand, chromosome 7 had the fewest *NRTs* genes, with only two genes identified (*NPF7.9* and *NPF8.5*). Members of the same subfamily were often distributed on multiple chromosomes. For example, we observed that the 23 *NPF8* genes were distributed across six different chromosomes, while the seven *NRT2* genes existed across six different chromosomes as well. Nevertheless, we also detected several *NPF*, *NRT2* and *NRT3* gene clusters in close genomic regions.

### 2.4. Gene Structure and Protein Domain Analysis of NPF, NRT2 and NRT3 Genes

**Figure 3:**
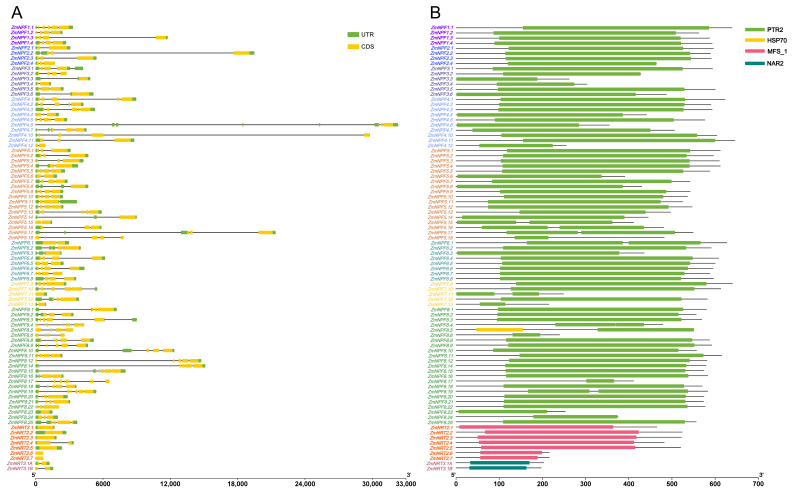
Schematic diagram of gene structure and functional protein domain of *NRT* genes in maize. (**A**) Gene structure. The green boxes, yellow boxes and gray lines represent the UTR, CDS and introns, respectively. (**B**) Protein domain of *NRT* genes. The protein domains are represented with a box. Box size indicates the length of the domain. The gray lines represent the non-functional sequences.

In order to examine variations in the structures of *NRTs* genes, we utilized the Visualize Gene Structure (Basic) module of TBtools [23] to visualize their exon and intron structures. As depicted in Figure 3A, genes belonging to the same subgroup exhibited similar exon-intron structures. Notably, nearly all *NPF* genes contained intron structures, with their coding sequences being interrupted by 1–5 introns. In contrast, except for *NRT2.4*, all *NRT2* genes featured a single exon and lacked introns.

We used Pfam and the Gene Structure View (Advanced) modules of TBtools [23,24] to annotate functional domains in the proteins encoded by *NRTs* genes, as illustrated in Figure 3. The proteins encoded by *NPF*, *NRT2*, and *NRT3* genes contained distinct domains, namely PTR2, MFS_1, and NAR2, respectively (Figure 3B). Notably, an HSP70 domain was identified in the protein sequence of NPF8.5.

### 2.5. Putative Cis-Acting Regulatory Elements (CREs) in the Promoters of NPF, NRT2 and NRT3 Genes

**Figure 4:**
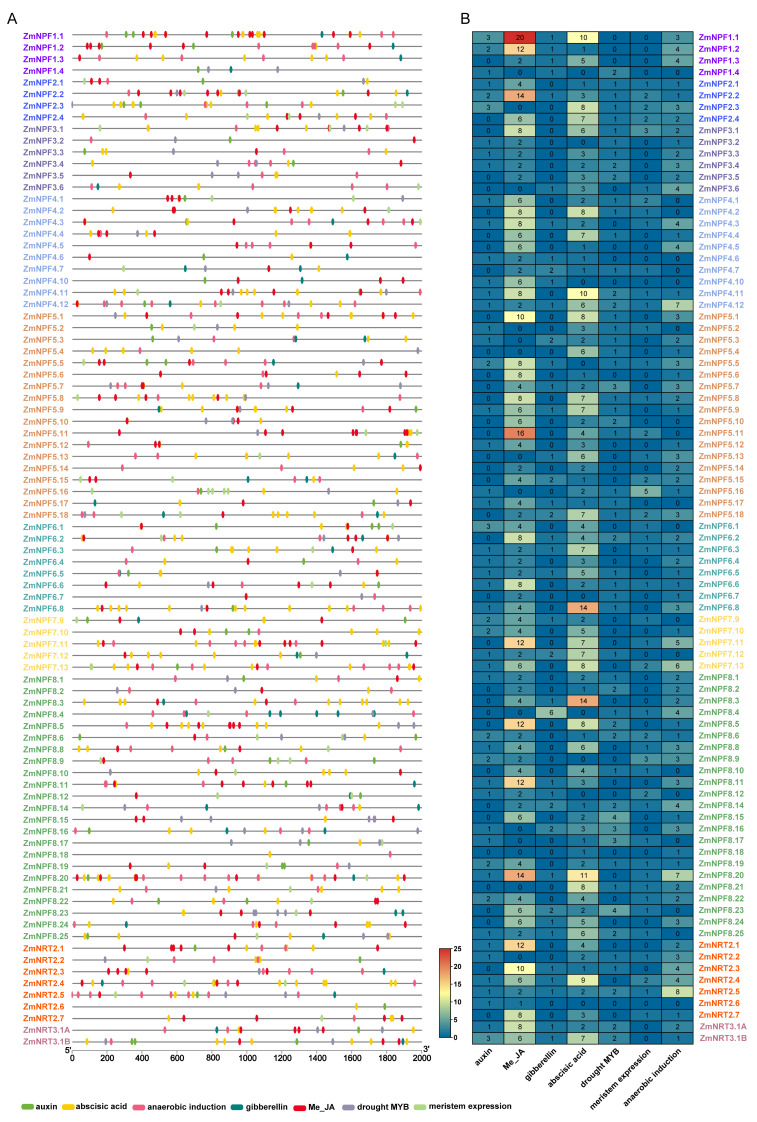
Analysis of *cis*-acting regulatory elements in the promoters of *NRT* genes in maize. (**A**) Schematic diagram of *cis*-elements in the promoters of *NRT* genes. (**B**) Frequency of representative *cis*-acting elements in the promoters of *NRT* genes.

To investigate the biological functions of *NRTs* genes, we conducted a thorough analysis of *cis*-acting elements by extracting the 2.0-kb fragment upstream of the transcription start site in the promoter region of each gene. Our analysis revealed a diverse array of functional elements within the promoter regions of *NRTs* genes, including light-responsive elements, hormone-responsive elements, anaerobic-responsive elements, and meristem expression elements (Figure 4). Among these elements, the Me-JA responsive and abscisic acid-responsive elements were found to occur at a higher frequency than other elements across all subfamilies. Particularly noteworthy was the *NRT3* subfamily, which exhibited the highest number of auxin and abscisic acid-responsive elements, while the *NRT2* subfamily displayed the lowest occurrence of these elements. Furthermore, the *NRT3* subfamily displayed the lowest frequency of meristem expression and anaerobic responsive elements, while the *NPF* and *NRT2* subfamilies had similar higher frequencies. Interestingly, the frequency occurrence of the *NRT2* subfamily’s response to gibberellin and drought was located in the middle.

### 2.6. Co-Functional Network Analysis of NPF, NRT2 and NRT3 Genes

To identify candidate genes that potentially interact with *NRTs* genes to regulate nitrogen assimilation or transportation, we employed the 87 *NRTs* genes as guide genes to construct a co-functional network using MaizeNet [25]. Among all 87 guide genes, no candidate genes were obtained for 15 guide genes including *ZmNPF1.3*, *ZmNPF3.3*, *ZmNPF4.2*, *ZmNPF4.4*, *ZmNPF4.11*, *ZmNPF5.18*, *ZmNPF7.11*, *ZmNPF7.13*, *ZmNPF8.4*, *ZmNPF8.6*, *ZmNPF8.17*, *ZmNRT2.4*, *ZmNRT2.6*, *ZmNRT2.7* and *ZmNRT3.1A*. A total of 762 candidate genes were obtained for the remaining 72 genes.

**Figure 5:**
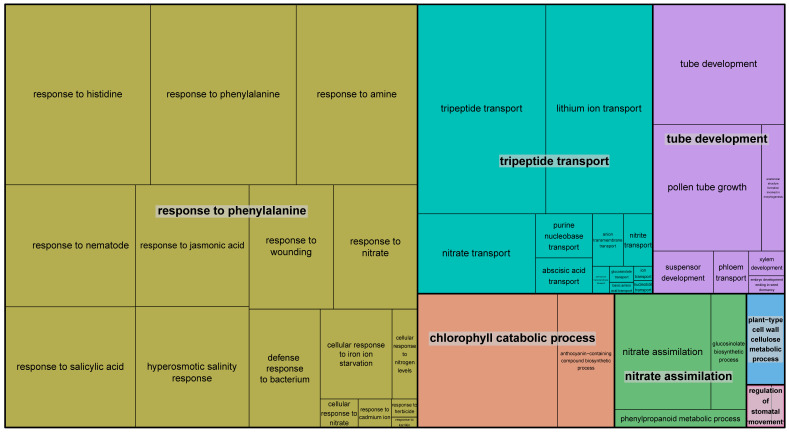
The TreeMap visualization of enriched GO terms associated with *NPF/NRT* genes and their interacting genes. The size of the rectangles indicates the number of genes involved in the corresponding biological progress.

Subsequently, we conducted a GO enrichment analysis utilizing both *NRTs* genes and their candidate interacting genes, employing the geneset analysis functionality implemented in MaizeNet. Enriched GO terms were visualized by Revigo with default parameters. This analysis revealed a significant enrichment of genes involved in tripeptide transport, tube development, nitrate assimilation, chlorophyll catabolic process, and phenylalanine response in the co-functional network of *NRTs* genes (Figure 5). Specifically, *NPF* genes and their interacting genes were mainly involved in the response to biological hormones, bacterium, wounding and chlorophyll catabolic process. On the other hand, *NRT2* genes and their interacting genes played significant roles in various biological processes such as lateral root development, cellular response to nitrate, nitrite transport, transmembrane transport, and response to Karrikin.

### 2.7. Spatial and Temporal Expression Analysis of NPF, NRT2 and NRT3 Genes

**Figure 6:**
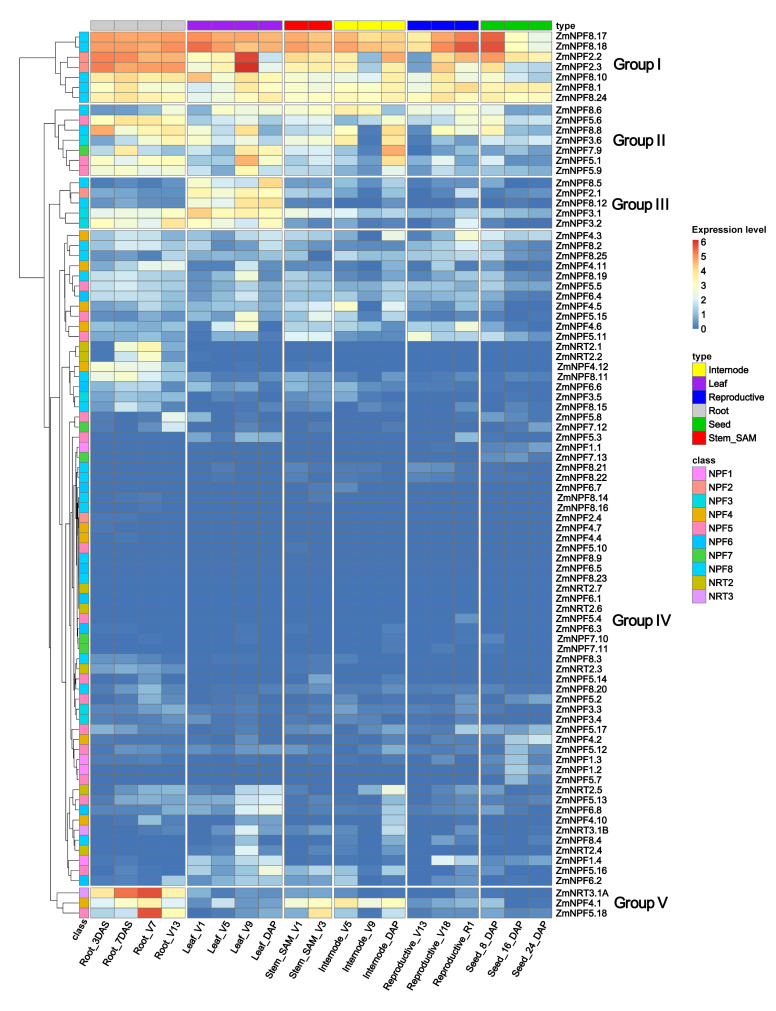
Expression profile of *NRT* genes. Gene expression data were normalized for each gene and are shown as log2- transformed data of normalized data + 1. Each column represents a tissue sampled at specific developmental stage, while each row represents a *NRT* gene. The subfamily of all *NRT* genes are indicated by colored boxes on the left. Tissues are represented by colored boxes on the top.

To gain insights into the spatiotemporal expression patterns of *NRTs* genes in maize, we extracted their gene expression data of different tissues/stages from qTeller (Table S2) [26]. The tissues were categorized into six groups, including roots, leaves, stem and shoot apical meristem (SAM), internode, reproductive tissues, and seeds. All *NRTs* genes were clustered into five distinct groups based on their expression patterns (Figure 6). Group I comprised five *NPF8* genes (*NPF8.17*, *NPF8.18*, *NPF8.10*, *NPF8.1* and *NPF8.24*) and two *NPF2* genes (*NPF2.2* and *NPF2.3*), which exhibited high expression levels in almost all tissues. In Group II, *NPF* genes were expressed at a moderate level across all tested tissues. Group III genes displayed relatively high expression levels in leaves, while their expression in seeds, reproductive tissues, internodes, and stems was lower. Group IV was the largest group, with the majority of genes showing low expression levels and distinct tissue-specific expression patterns. Among these genes, *ZmNRT2.1* and *ZmNRT2.2* were specially expressed in roots. Group V was the smallest, containing only three genes—*ZmNRT3.1A*, *ZmNPF4.1*, and *ZmNPF5.18*—all of which showed high expression levels primarily in roots.

### 2.8. Expression Profiles of NRTs Genes in Response to Nitrogen Starvation and Nitrate Resupply

**Figure 7:**
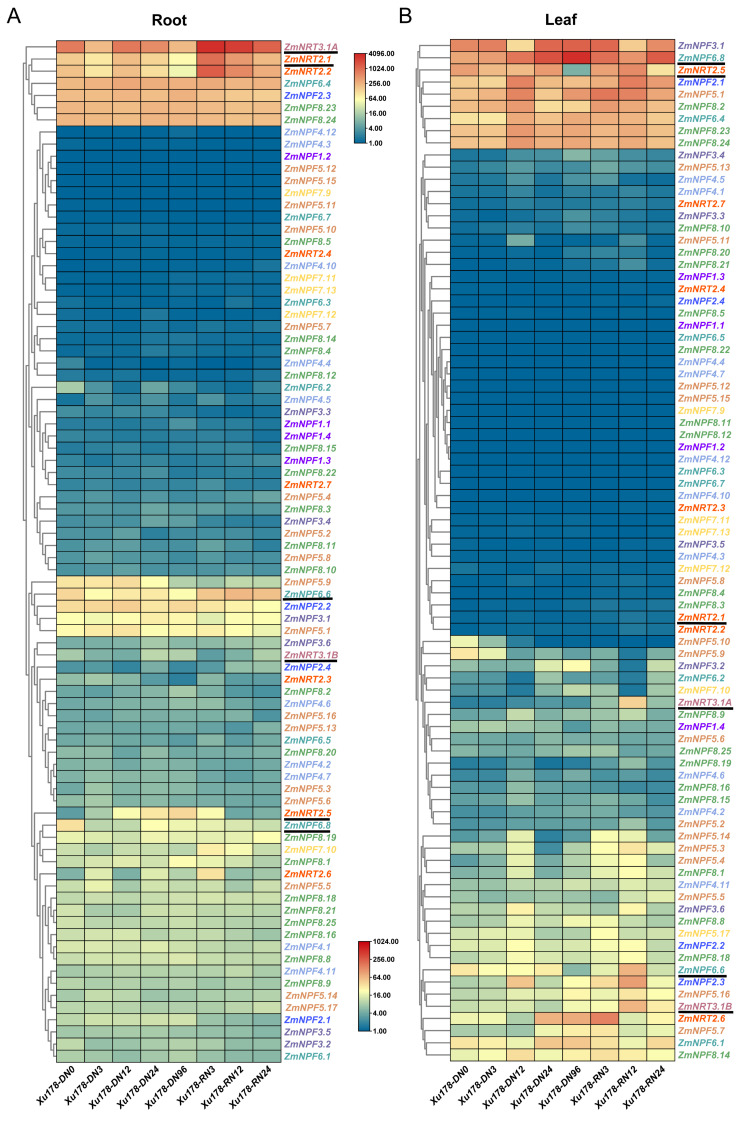
Expression profile of *NRT* genes in the root and leaf of Xu178 in response to nitrogen starvation and nitrate resupply. (**A**) Each column represents the root of Xu178 sampled at a specific time point. (**B**) Each column represents the leaf of Xu178 sampled at a specific time point. DN3, treated with nitrogen-deprived (DN) nutrient solution for 3 h. RN3, nitrogen resupply (RN, 4 mM NO_3_^−^) for 3 h. Representative genes of multiple groups are highlighted with underlines.

RNA sequencing was conducted using the maize inbred line Xu178 to investigate the expression patterns of *NRTs* genes in response to nitrogen levels (Section 4, Table S3). The third leaves and roots of Xu178 seedlings were sampled at various time points during nitrogen starvation and nitrate resupply. More than 20 million clean reads were obtained for each sample. Based on the nitrate-response expression profiles in roots, *NRTs* genes were classified into four distinct groups (Figure 7). Genes in group I demonstrated the highest expression levels among all time points, indicating their significant roles in nitrate uptake in maize. Notably, *NRT2.1*, *NRT2.2* and *NRT3.1A* displayed decreased expression levels during prolonged nitrogen starvation but exhibited a rapid increase in expression after nitrate resupply, highlighting their responsiveness to changes in nitrogen levels. The similar expression trend of *NRT2.1/2.2* and *NRT3.1A* indicated that *NRT3.1A* might be involved in nitrate transport activity of *NRT2.1/2.2* in maize. Genes in group II had low expression levels in roots and exhibited no evident response to changes in nitrogen levels. Group III comprised five *NPF* genes with relatively high expression levels, and among them, *NPF6.6* exhibited a clear nitrate response, similar to *NRT2.1/2.2*. Finally, genes in group IV had moderate expression levels and were found to be responsive to nitrogen starvation and nitrate resupply. For instance, the expression of *NRT2.5* was upregulated with prolonged nitrogen starvation and reduced after nitrate resupply. Its expression level was restored to DN0 when the recovery of nitrate supply was 12 h (RN12).

The expression patterns of *NRTs* genes in leaves, in response to nitrogen levels, differed significantly from those in roots and were classified into five groups. Genes in group I displayed the highest expression levels in leaves, which varied with nitrogen starvation and resupply. Notably, this group included *NPF8.23*, *NPF8.24*, and *NPF6.4* genes, which were also highly expressed in roots. The expression of *NPF6.8* in leaves continued to increase with prolonged nitrogen starvation, peaking at DN96, but decreased after nitrate resupply, exhibiting a response pattern similar to *NRT2.5* in roots. However, the response pattern of *NRT2.5* to nitrogen levels in leaves was opposite to that in roots, as its expression continuously decreased under nitrogen starvation stress and rapidly increased after nitrate resupply. Approximately half of the *NRTs* genes were clustered in group II, with low or no expression in leaves, and they did not significantly respond to changes in nitrogen levels, including most *NRT2* genes (except *NRT2.5* and *NRT2.6*). *NRT3.1A*, in group III, showed low expression levels during nitrogen starvation but increased after nitrate resupply, reaching its peak at RN12. Genes in groups IV and V had moderate to high expression levels in leaves, exhibiting a clear response to changes in nitrogen levels. The response pattern of *NPF6.6* was similar to that of *NRT2.5*, as its expression decreased during nitrogen starvation but was upregulated after nitrate resupply. These results imply that different *NRTs* are responsible for nitrate uptake and transport in the roots and leaves of maize. Moreover, their responses to nitrogen starvation and nitrate supply significantly vary between these two tissues, highlighting the intricate and tissue-specific regulation of nitrogen utilization in maize.

### 2.9. NRTs Expression Patterns Exhibit Variations among Diverse Inbred Lines with Different Nitrate Uptake Rates

**Figure 8:**
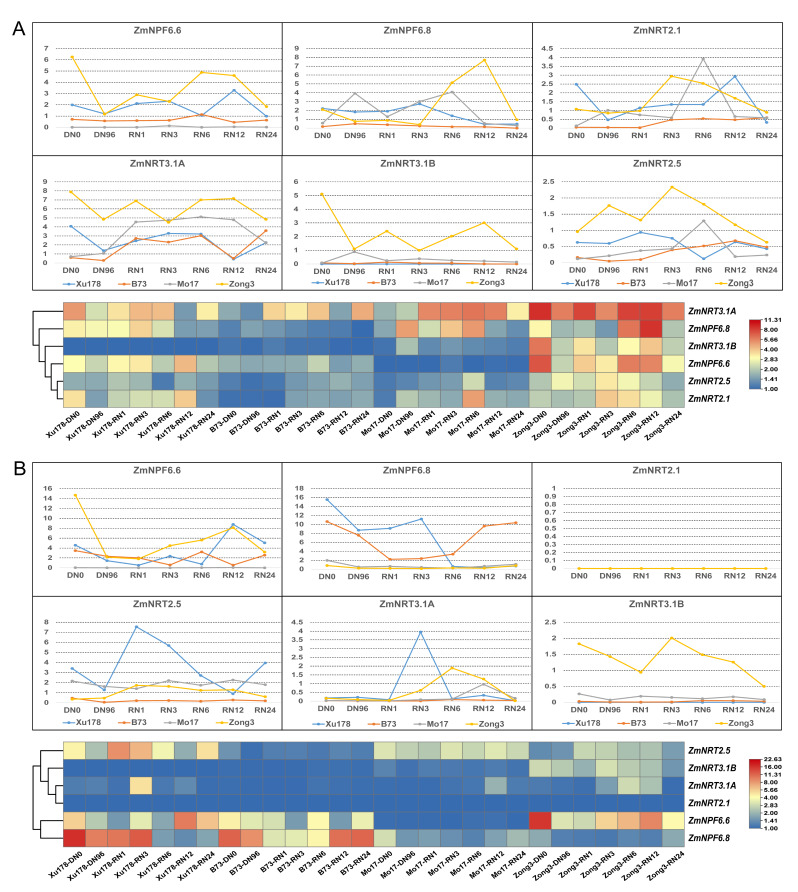
Expression profile of six *NRT* genes in four maize inbred lines in response to nitrogen starvation and nitrate resupply. (**A**) Each column represents the root of four maize inbred lines sampled at specific time point. (**B**) Each column represents the leaf of four maize inbred lines sampled at specific time point. DN3, treated with nitrogen-deprived (DN) nutrient solution for 3 h. RN3, nitrogen resupply (RN, 4 mM NO_3_^−^) for 3 h.

To explore the differences in the response patterns of *NRT* genes among different maize inbred lines, we focused on six key genes (*NPF6.6*, *NPF6.8*, *NRT2.1*, *NRT2.5*, *NRT3.1A* and *NRT3.1B*) that showed varying nitrate response patterns based on RNA-seq results. These genes were subjected to RT-qPCR analysis to assess their nitrate response patterns in four maize inbred lines (Figure 8). We observed a remarkable difference in nitrate uptake rates among the four maize inbred lines (Section 4, Figure S1). Particularly, the inbred line B73 exhibited significantly higher nitrate uptake efficiency compared to the other inbred lines. Furthermore, the time points at which the four inbred lines reached their maximum uptake efficiency varied.

The expression of *NRTs* genes in the roots of the four inbred lines exhibited varied responses to nitrate supply, with Zong3 showing the highest gene expression level (Figure 8A). *ZmNRT2.1* and *ZmNRT2.5* exhibited similar expression patterns in response to changes in nitrate levels in the roots of all four inbred lines. *ZmNRT2.1* had the lowest expression at RN1h in all four inbred lines, but the time at which it reached its peak differed. It reached its highest peak at RN3h in B73 and Xu178, while in Zong3 and Mo17, it peaked at RN6h. In contrast to *ZmNRT2.1*, the expression level of *ZmNRT2.5* peaked at RN1h in all four inbred lines and then decreased with prolonged nitrate resupply time. The expression level of *ZmNPF6.6* in Zong3 decreased at RN3h, while in the other three inbred lines, it decreased at RN6h. The expression patterns of *ZmNPF6.8* in response to changes in nitrate levels were alike in B73 and Xu 178, but they exhibited a significant reduction at RN1h in Zong3 and Mo17. Regarding *ZmNRT3.1A*, its expression in the four inbred lines showed similar response patterns to changes in nitrate levels, with the differences mainly reflected in the expression levels. On the other hand, the expression of *ZmNRT3.1B* in Mo17, B73, and Xu178 was consistent, contrasting with its expression in Zong3.

In leaves (Figure 8B), the response patterns of the six key *NRTs* genes to changes in nitrate levels in the four inbred lines were similar, which were significantly different from the results observed in roots. The highest expression levels of these genes were observed in Zong3, consistent with the results in roots.

## 3. Discussion

### 3.1. Diversity in Structure and Function of NRTs Gene Families

Nitrate transporter genes play a crucial role in the uptake and transportation of nitrate in plants, significantly contributing to improving plant nitrogen efficiency (NUE). In addition to *Arabidopsis thaliana*, studies have been carried out on the *NRTs* gene family in crops such as rice, barley and wheat [3,27,28,29]. While previous research had identified *NRTs* family members in maize through homologous sequence alignment [3] and analyzed expression patterns and electrophysiological characteristics of some *NRTs* genes in different tissues and developmental stages [19,21], a systematic analysis of *NRTs* family members, including genetic evolution, gene structure, and functional element analysis, has been lacking. Moreover, no analysis has been conducted on nitrate response and its contribution to improving crop NUE and yield.

In our study, we used the newly published and more accurate maize genome sequence to identify 78 *NPF* genes, seven *NRT2* genes, and two *NRT3/NAR2* genes, through conserved functional domains analysis of *NRTs*. The distribution of *NPF* and *NRT2* family genes on chromosomes was uneven, and genetic evolution analysis identified eight subfamilies of *NPF* family genes, similar to *Arabidopsis thaliana* and rice [3].

*NPF* family members were found to have a range of two to five introns, while all *NRT2* genes, except for *NRT2.4*, were intron less, indicating the conservation of *NRT2* gene during evolution. *Cis*-acting elements play critical roles in gene response to environment changes and the regulation of their expression in various biological processes [30] The promoter sequences of *NRTs* family members contain a diverse range of *cis*-acting elements, with abscisic acid responsiveness and Me-JA responsiveness elements being particularly abundant. Similarly, *NRTs* genes of *Arabidopsis thaliana* and rice contain various plant hormones (IAA, ABA, JAs, and/or GAs) *cis*-acting elements [31], providing the foundation for *NRTs* genes to respond to stresses such as drought, cold, heat, UV, and salt.

In *Arabidopsis thaliana*, *AtNPF6.3* was highly induced by IAA treatment under low nitrogen conditions [32], *AtNPF2.10* was upregulated by Me-JA treatment, thereby accelerating the transport of gibberellin [33], while *AtNPF3.1* was transcriptionally regulated by both GA and ABA [34,35]. Additionally, auxin may also mediate nitrogen uptake and transport, which is derived from the changes in the expression of many *NRTs* genes under IAA treatment [36]. In rice, it has been reported that *OsNRT2.4* is up-regulated by exogenous auxin and JA [37].

We integrated and analyzed publicly available transcriptome data from maize and found that genes belonging to the *NPF* and *NRT2* families displayed varying degrees of responsiveness to stress conditions (Figure S2). According to the analysis of *cis*-acting regulatory elements of *NRTs* genes (Figure 4), *NPF8.15* and *NPF8.23* were found to respond to drought stress, *NPF1.1* responded to Me-JA, *NRT2.4* and *NRT3.1B* responded to ABA. Function analysis of the *NRTs* family members revealed their broad functional scope, with *NPF* genes potentially involved in even more diverse physiological processes.

### 3.2. Variation in the Expression Patterns of NRTs Genes

Gene expression patterns in plant tissues and developmental stages offer valuable insights into potential gene functions. Our integrative analysis of published RNA-seq data revealed specific patterns for a few *NRTs* genes, indicating their crucial roles in plant and organ development. In roots, seven genes (*ZmNPF8.17*, *ZmNPF8.18*, *ZmNPF2.2*, *ZmNPF2.3*, *ZmNPF8.10*, *ZmNPF8.1*, and *ZmNPF8.24*) showed consistently high expression levels that were minimally affected by developmental stages. The functions of some homologous genes in rice have been analyzed. For example, *OsNPF8.1* have been reported to contribute to dimethyl arsenate accumulation in rice grains [38], and *OsNPF2.2* plays a role in unloading nitrate from the xylem and influencing nitrate transport and plant development [9]. Along with the seven genes mentioned above, genes *ZmNRT3.1A*, *ZmNPF4.1*, and *ZmNPF5.18A* also exhibit high expression levels in the roots, however, their expression levels are significantly influenced by the developmental period. The expression pattern of *NRT2.1* and *NRT2.2* genes in roots resembled that of *NRT3.1A*. Several genes have been identified that are involved in root nitrate uptake in *Arabidopsis thaliana*, including *AtNRT2.1/2.2/2.4/2.5*, *AtNPF6.3*, and *AtNPF4.6* [32,39,40,41]. Similarly, in rice, genes such as *OsNRT2.1/2.2/2.4* and *OsNPF2.4/6.5/8.9* have been found to be involved in root nitrate uptake [7,8,13,37,42,43]. Research conducted in maize has revealed that *ZmNPF6.6* plays a role in nitrate transport [21]. The *NRT* genes, which exhibit high expression levels in roots, are likely to be involved in the process of nitrate uptake by the roots.

In leaves, *NRT* gene expression is more markedly influenced by developmental stages and organ tissues. For instance, the expression levels of *ZmNPF2.2* and *ZmNPF2.3* genes peak at the V9 stage, whereas the expression of *ZmNPF6.8*, *ZmNRT2.5*, and *ZmNRT3.1B* are higher during the pollination period than before, and their expression in leaves are considerably higher than that in roots. Following nitrate uptake by the plant roots, specific *NRTs* genes are responsible for nitrate transport and distribution in leaves. For example, the *OsNPF2.4* gene in rice plays a role in nitrate redistribution in leaves, while in *Arabidopsis thaliana*, *AtNPF1.1/1.2/2.13*, *AtNRT2.4*, and *AtNRT2.5* genes regulate nitrate redistribution between sources and sinks in leaves.

Adequate nitrate is crucial for seed development in plants, and specialized *NRT* genes are responsible for nitrate transport to the grains. For instance, the *OsNPF2.4* gene in rice regulates seed vigor [13], while overexpression of the *TaNRT2.5* in wheat can increase nitrate accumulation, seed vigor, and yield [44]. In *Arabidopsis thaliana*, *AtNRT2.7* controls the nitrate content in grains [45]. In maize, we observed that *ZmNRT2.5* had peak expression in maize seeds at 2.5 days after pollination, while the expression of *ZmNPF8.17* and *ZmNPF8.18* genes was highest at eight days after pollination but decreased as pollination time was delayed. However, it remains unclear whether these two genes are involved in nitrate transport during seed development.

In internodes, *NRTs* gene expression varied with developmental stage. *ZmNRT2.5*, *ZmNPF5.1*, and *ZmNPF7.9* exhibited significantly higher expression during pollination than during vegetative growth, while *ZmNPF2.2* and *ZmNPF2.3* had lower expression levels at the V9 stage than at other stages. *ZmNPF6.6* expression gradually decreased during growth and during development. In rice, *OsNPF2.4* and *OsNPF6.5* were responsible for nitrate transport from roots to shoots, suggesting that internode genes primarily participate in nitrate transport, and their expression varies with developmental stage.

### 3.3. Maize NRT Gene Expression Is Regulated by Nitrate Levels and Genetic Background

To investigate the impact of nitrogen levels on the expression of *NRTs* genes in maize, RNA-seq was performed to analyze the expression patterns of different *NRTs* genes in the roots and leaves of maize under nitrogen-deprived and nitrogen-resupply conditions. The results showed that nearly half of the *NRTs* genes in roots did not exhibit significant changes in response to alternations in nitrogen levels. However, *ZmNRT2.1*, *ZmNRT2.2*, and *ZmNRT3.1A* displayed high expression levels in the roots and showed a considerable response to nitrate levels. During an extended period of nitrogen deprivation, the expression of *ZmNRT2.1* declined but significantly increased after three hours of nitrogen resupply, and then decreased once again with continued nitrogen supply. The NRT2s are high-affinity nitrate transporters, and their transport activity frequently requires the support of NRT3. A previous study conducted in *Arabidopsis thaliana* has demonstrated that the transcript abundance of *AtNRT2.1* was significantly correlated with the high-affinity nitrate influx after the provision of nitrate to nitrate-deprived plants [46]. Increased expression of *OsNRT2.1* was associated with enhanced nitrogen-use efficiency and nitrate-dependent root elongation in rice [42,43]. Transcript levels of *ZmNRT2.1* and *ZmNRT2.2* were observed to be correlated with high-affinity root nitrate uptake capacity and also nitrogen availability [19]. In both nitrogen-starved and nitrogen-resupply states, *ZmNPF6.6* displayed higher expression, with a significant increase observed after nitrogen resupply (Figure 7A). Studies conducted on *Arabidopsis thaliana* and rice revealed that the ZmNPF6.6 homologous, AtNPF6.3, and OsNPF6.5, function as double-affinity nitrate transporters [7,32]. Increased expression of *OsNRT1.1B* leads to an accumulation of N in plants, promoting rice growth under low N conditions [47]. In contrast, *ZmNRT2.5* in roots exhibited a different response pattern with its expression continuing to increase under nitrogen-starved conditions and returning to pre-starvation condition levels following a prolonged period of nitrogen-resupply (Figure 7A). This indicates that *ZmNRT2.5* may play an important role in mediating nitrate uptake by plants under long-term low nitrogen stress. It has demonstrated that *AtNRT2.5* displayed the highest expression level in roots among the seven *Arabidopsis thaliana NRT2* genes under long-term nitrogen starvation stress [41]. Overexpression of the homologous gene *OsNRT2.3a*, which is identical to *NRT2.5* in rice, significantly improves nitrogen use efficiency and increases the yield of rice under low nitrogen conditions [48]. The homologous gene *TaNRT2.5* in wheat is expressed in the embryo and shell and plays an important role in the accumulation of nitrate in seed [44].

The response patterns of genes in leaves differ significantly from those in roots, with most of the genes in leaves showing no significant response to nitrate levels. In our study, the expression level of *ZmNPF6.8* in leaves is upregulated with the extension of nitrogen starvation time (Figure 7B). The previous study found that *ZmNPF6.8* have a higher expression pattern in old leaves than in root or young leaves during vegetative and reproductive development stages [20]. According to the existing results, it is speculated that the *ZmNPF6.8* gene is responsible for leaf nitrogen remobilization. *ZmNRT2.5* exhibits an expression pattern opposite to that in roots, increasing the expression level in shoots when nitrogen is sufficient and decreasing the expression when nitrogen is deficient (Figure 7B). In rice, the corresponding gene *OsNRT2.3a* has a specific role in transporting nitrate from the roots to the shoots [18]. In addition to *ZmNRT2.5*, *ZmNPF6.6* also has relatively high expression levels in both roots and leaves. Previous research has reported that nitrate-induced upregulation of *ZmNPF6.6* in leaves is rapid and homologous genes, such as *AtNPF6.3*, have been shown to enhance expression in shoots and improve growth under nitrogen deficiency stress [49]. This suggests that *ZmNPF6.6* responds quickly to external nitrate in leaves, promoting growth under nitrogen deficiency stress.

To investigate the relationship between *NRTs* gene expression levels and plant nitrate uptake efficiency, four inbred maize lines with varying nitrate uptake efficiencies were selected to analyze the expression patterns of different *NRTs*. The results indicated that the expression patterns of candidate *NRTs* (*NRT2.1*, *NRT2.5*, *NPF6.6*, *NPF6.8*, *NRT3.1A*, *NRT3.1B*) in response to nitrate changes differed among different genetic backgrounds. Studies have shown that different genotypes of the same gene contribute differently to the phenotype of the plant. It has been demonstrated that variations in the *NRT1.1B (OsNPF6.5)* affect nitrate use efficiency in different subspecies of rice. *OsNRT1.1B-indica* transgenic plants exhibited better growth performance and greater NUE than *OsNRT1.1B-japonica* transgenic plants [7]. Tang et al. (2019) identified a rare natural allele, *OsNPF6.1^HapB^*, which enhances nitrate uptake and improves NUE in rice [17]. The findings imply that identifying and harnessing beneficial allelic variations of key *NRTs* genes in crops can be a promising approach to enhancing crop NUE through genetic means. Our findings reveal variations in the response strength of *NRTs* to nitrate among distinct inbred lines of maize, suggesting the presence of allelic diversity in *NRTs* across different materials that may impact the NUE of maize.

## 4. Materials and Methods

### 4.1. Identification of NRTs Genes in Maize

To identify all the *NPF* genes in the maize genome, we performed a protein sequence alignment of 53 *NPF* genes annotated in *Arabidopsis thaliana* against all protein sequences of the maize B73 genome (version 5, http://www.maizegdb.org (accessed on 20 June 2022)) using BLASTP [3,50,51]. Candidate *NPF* genes were selected by filtering the BLASTP results according to specific criteria (e-value ≤ 1 × 10^−3^, identity ≥ 30%, query coverage ≥30%). We then used HMMER (v3.0) to search for the “Proton-dependent oligopeptide transporter family” (IPR000109) domain in the protein sequences encoded by all candidate *NPF* genes, and excluded proteins without this domain (e-value ≤ 1 × 10^−5^) [52]. The remaining genes were identified as *NPF* genes in maize. The same approach was used to identify *NRT2* and *NRT3* genes in the maize genome.

### 4.2. Phylogenetic Analysis

We performed a multiple sequence alignment using the protein sequences encoded by the 78 *NPF*, seven *NRT2* and two *NRT3* genes using the MUSCLE algorithm implemented in MEGA7 [53]. A phylogenetic tree was subsequently constructed based on the alignment results using the neighbor-joining (NJ) method implemented in MEGA7 with 1000 bootstrap replicates. The tree was further enhanced using the iTOL software (https://itol.embl.de/ (accessed on 17 August 2022)) [54].

### 4.3. Chromosomal Localization of NPF, NRT2 and NRT3 Genes

We obtained the chromosomal locations of the 87 *NPF/NRT1*, *NRT2* and *NRT3* genes in the maize B73 genome (V5) from MaizeGDB (http://www.maizegdb.org (accessed on 20 Jun 2022)), and subsequently visualized them by creating circular plots using shinyCircos (https://venyao.xyz/shinyCircos/ (accessed on 10 July 2022)) [55].

### 4.4. Gene Structure Analysis

The exon/intron structures of the *NPF*, *NRT2*, and *NRT3* genes were extracted from the gene annotation file of the maize B73 genome (V5), and visualized using the gene structure view module (advanced) of TBtools [23].

### 4.5. Identification of Putative Cis-Acting Regulatory Elements

For the identification of putative *cis*-acting regulatory elements, we obtained a 2.0-kb genomic sequence upstream of the transcription start sites for all *NPF*, *NRT2*, and *NRT3* genes in the maize B73 reference genome (V5). These sequences were analyzed using Plant CARE (http://bioinformatics.psb.ugent.be/webtools/plantcare/html/ (accessed on 15 November 2022)) [56].

### 4.6. Functional Domain Analysis

To annotate functional domains in all *NPF*, *NRT2* and *NRT3* genes, we utilized Pfam and visualized the domains using the “Visualize Domain Pattern (from Pfam search)” module of TBtools [23,24].

### 4.7. Expression Analysis with Public RNA-Seq Data

The expression levels of *NPF*, *NRT2*, and *NRT3* genes in various tissues of B73 at diverse developmental stages were retrieved from qTeller (http://qteller.maizegdb.org (accessed on 20 March 2023)) [26].

### 4.8. Plant Materials and Growth Conditions

Surface sterilization of seeds from four maize inbred lines (Xu178, Zong3, Mo17, and B73) was carried out using a 10% (*v*/*v*) hydrogen peroxide solution for 30 min, followed by rinsing with distilled water and soaking in distilled water at room temperature for 12–16 h. After germinating in distilled sand, uniform seedlings were transferred to plastic containers and grown in a half-strength nutrient solution for two days, with the residual endosperm removed before transfer. Subsequently, the seedlings were transferred to a full-strength nutrient solution with high nitrogen (HN, 4 mM NO_3_^−^). Once the third leaf emerged from the leaf collar (approximately 7 days cultured in HN solution), the uniform seedlings were transferred to nitrogen-deprived (DN) nutrient solution for 96 h (DN96), followed by nitrogen resupply (RN, 4 mM NO_3_^−^) for 24 h (RN24). Each treatment was conducted with three biological replicates. The full-strength nutrient solution consisted of 2 mM Ca(NO_3_)_2_, 0.75 mM K_2_SO_4_, 0.65 mM MgSO_4_·7H_2_O, 0.1 mM KCl, 0.3 mM KH_2_PO_4_, 0.025 mM K_2_HPO_4_·3H_2_O, 0.1 mM Fe-EDTA, and micronutrients (1 µM H_3_BO_3_, 1 µM MnSO_4_·H_2_O, 0.01 µM CuSO_4_·5H_2_O, 1 µM ZnSO_4_·7H_2_O and 0.05 µM (NH_4_)_6_Mo_7_ O_24_·4H_2_O) at a pH of 6.0, as previously described by Guo et al. [28]. In the nitrogen-deprived treatment, 2 mM CaCl_2_ was used to compensate for the reduction in Ca^2+^ concentration.

### 4.9. Sample Collection for RT-qPCR and Transcriptome Sequencing

The third leaves and roots were collected at various time points after the onset of nitrogen deprivation and resupply, and all samples were immediately frozen in liquid nitrogen and stored at −80 °C. For RT-qPCR, samples of four maize inbred lines were collected at 0 h and 96 h after nitrogen deprivation, and 1 h, 3 h, 6 h, 12 h, and 24 h after nitrogen resupplied. For RNA sequencing, 48 samples of Xu178 were collected at 0 h, 3 h, 12 h, 24 h, and 96 h after nitrogen deprivation, and 3 h, 12 h, and 24 h after nitrogen resupplied.

### 4.10. RNA Extraction, cDNA Synthesis, and RT-qPCR

Total RNAs were extracted using Trizol (Invitrogen, Carlsbad, CA, USA) according to the manufacturer’s instructions. First-strand cDNAs were synthesized from the total RNA using HiScript II Q RT Super Mix for qPCR (+gDNA wiper) (Vazyme, Nanjing, China). Subsequently, RT-qPCR was conducted using the Eva Green 2X qPCR Master Mix-Low ROX Kit (abm) on the Quant Studio 3 System (Applied Biosystems of Thermo Fisher Scientific, Waltham, MA, USA), following the manufacturer’s protocol. The relative expression levels of the target genes were determined by normalizing them to the abundance of *ZmActin1* detected in the same sample and presented as 2^−ΔCT^. The RT-qPCR primers used are listed in Table S4.

### 4.11. Transcriptome Sequencing and Data Processing

The extracted total RNAs were initially subjected to a thorough quality assessment. Only the high-quality RNA samples were selected to create sequencing libraries, which were prepared using the Illumina TruSeq RNA Library Preparation Kit v2. Subsequently, these libraries underwent sequencing using 150-bp paired-end reads on the Illumina HiSeq X Ten (GSA (Genome Sequence Archive) database Accession Number: CRA012157).

For each set of RNA sequencing data, we first removed the adapters and low-quality reads using Fastp (version v0.20.1) [57] and then aligned clean reads to the maize B73 reference genome (version 5, http://www.maizegdb.org (accessed on 20 June 2022)) using STAR (version 2.7.5a) [58] with default parameters. Based on the alignment results, we then calculated the read counts for each gene using htseq-count from HTSeq (version 1.0) [59] and obtained their expression levels using edgeR (version 3.28.1) [60].

### 4.12. Determination of Nitrate Uptake Rate

To measure the nitrate uptake rate, we employed the depletion method with a sheltered plastic bowl. First, seedlings from four inbred lines were cultured in HN nutrient solution until the third leaf fully expanded. After 96 h of nitrogen starvation treatment, uniform seedlings were selected from each line, and their roots were submerged in an aerated uptake solution containing 1 mM NO_3_^−^. Samples were collected at various time intervals (0, 0.5, 1, 1.5, 2, 2.5, 3, 6, 9, 12, and 24 h), and the fresh weight of the root was recorded. The concentration of NO_3_^−^ was measured using the sulfuric acid salicylic method at 410 nm, employing a Thermo Scientific Microplate Reader. Kinetic parameters of NO_3_^−^ uptake in the root system, including Imax (maximum uptake rate), Km (the concentration of NO_3_^−^ ions in uptake solution when the uptake rate is equal to 1/2 Imax), and Cmin (the concentration of NO_3_^−^ ions in the uptake solution when the uptake rate is equal to zero), were calculated based on the curve y = ax^2^ + bx + c, where x is the concentration of NO_3_^−^ in uptake solution and y is the rate of NO_3_^−^ ions uptake per unit of fresh root weight, according to the formula Imax = (4ac − b^2^)/4a. The calculated results of the kinetic parameters are listed in Table S5.

## 5. Conclusions

Nitrate transporters play critical roles in facilitating the uptake, translocation, and distribution of nitrate in plants. Identification of key contributing *NRTs* can significantly improve crop grain yield and NUE. Our study consisted of a comprehensive analysis of all *NRTs* in maize, involving investigations into their phylogenetic relationships, gene structure, and expression patterns. By employing RNA-seq, we systematically assessed the response patterns of *NRTs* to variations in nitrate levels and analyzed the expression patterns of several potential *NRTs* candidates in diverse maize inbred lines. These results establish a foundation for deepening our understanding of the molecular functions of *NRTs* in maize and provide insights into the identification of crucial *NRTs* related to maize nitrogen utilization efficiency for promoting the development of nitrogen-efficient maize varieties.

## Data Availability

Data are contained within this article and Supplementary Material.

## Supplementary Materials

The supporting information can be downloaded at: https://www.mdpi.com/article/10.3390/ijms241612941/s1.
