# Supplementary material for: Genome-Wide Identification and Functional Analysis of Nitrate Transporter Genes (NPF, NRT2 and NRT3) in Maize
Source: Int J Mol Sci. 2023 Aug 18;24(16):12941. doi: 10.3390/ijms241612941 (PMC10454388; doi:10.3390/ijms241612941)
Supplement: Supplementary file 1 [file ijms-24-12941-s001.zip › Supplemental Figures.pptx]

## Slide 1
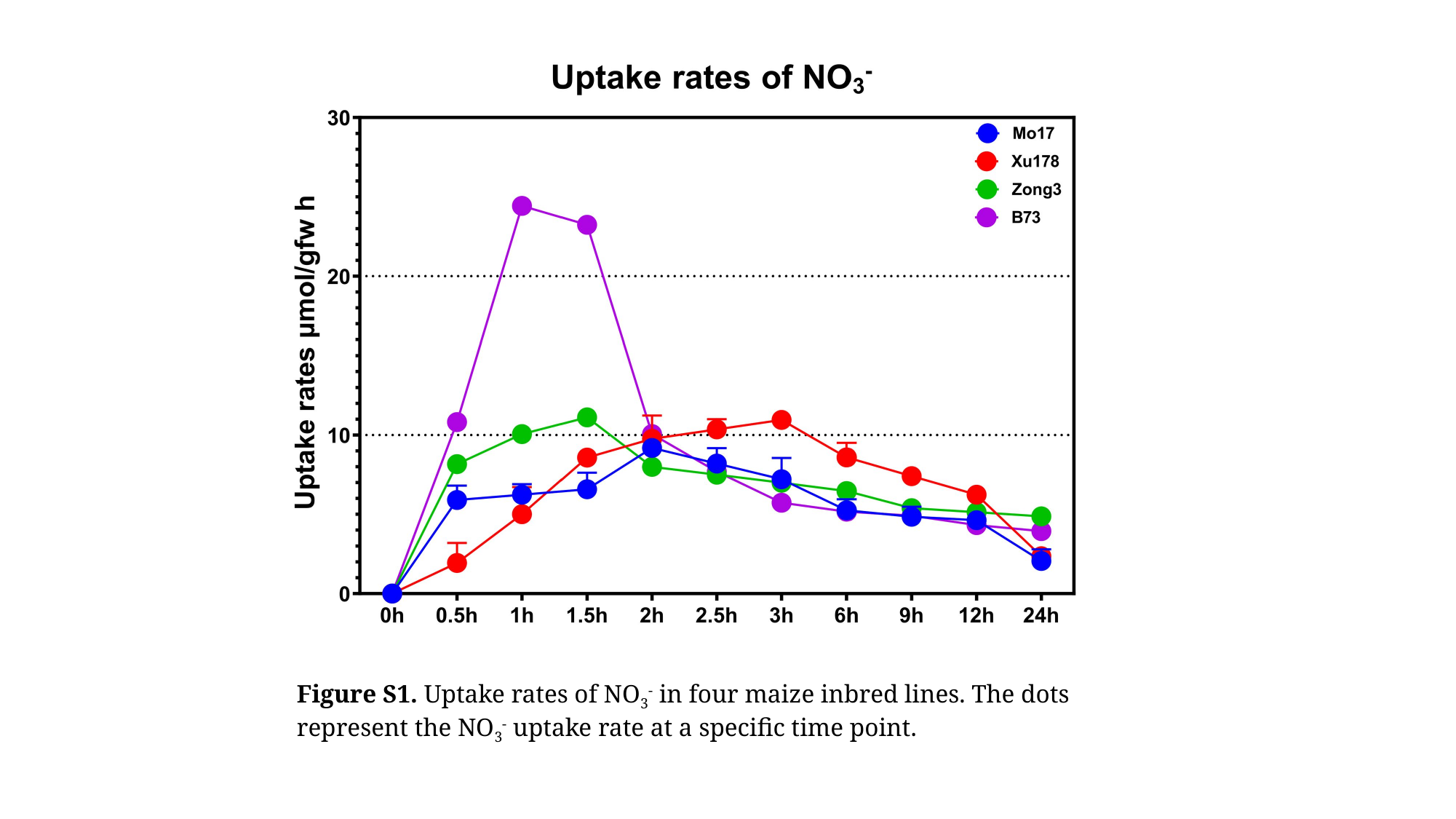

Figure S1. Uptake rates of NO3- in four maize inbred lines. The dots represent the NO3- uptake rate at a specific time point.

## Slide 2
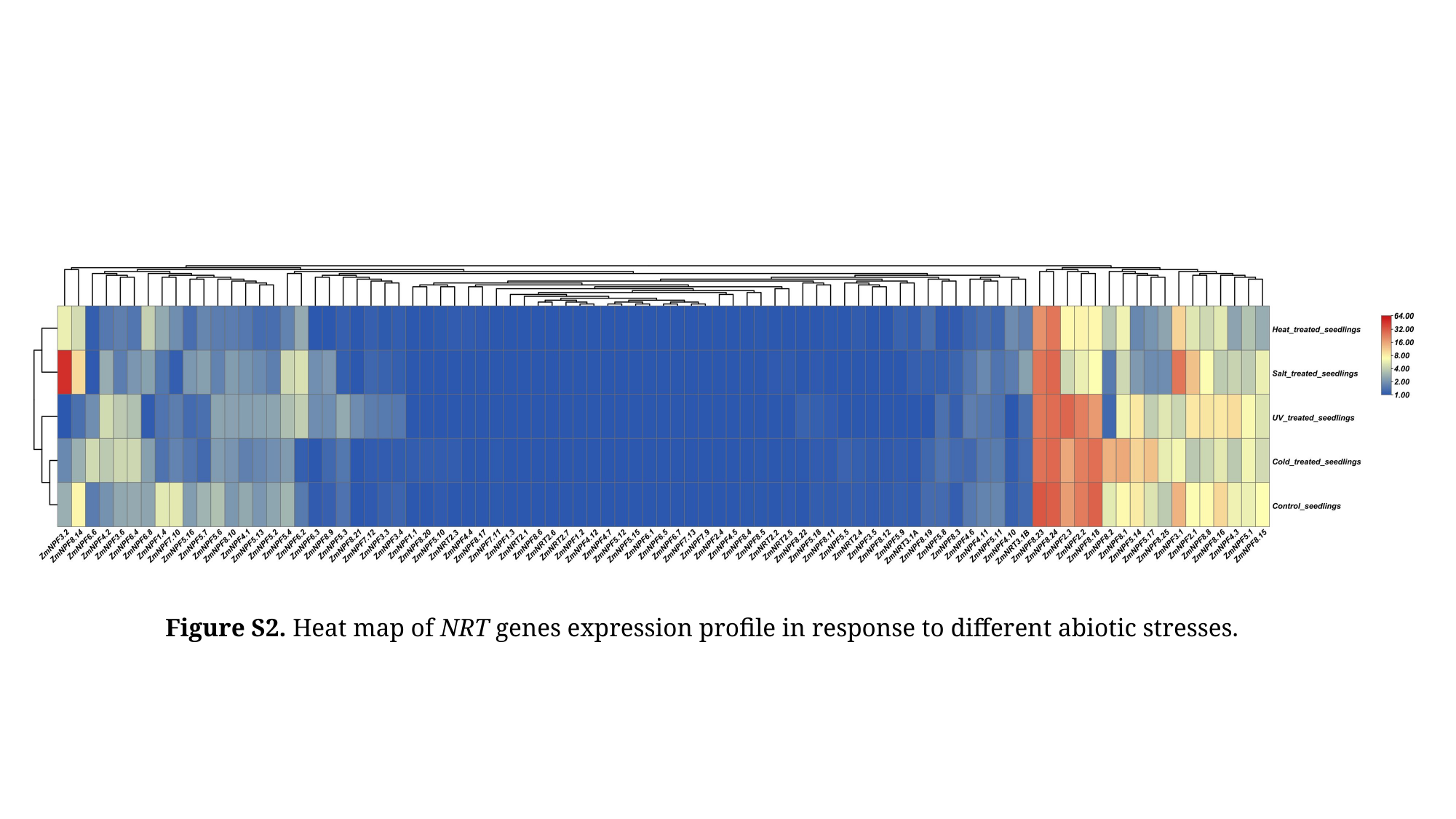

Figure S2. Heat map of NRT genes expression profile in response to different abiotic stresses.
